# Minimal internal limiting membrane peeling with ILM flap technique for idiopathic macular holes: a preliminary study

**DOI:** 10.1186/s12886-020-01505-x

**Published:** 2020-06-15

**Authors:** Zizhong Hu, Huiming Qian, Silvia Fransisca, Xunyi Gu, Jiangdong Ji, Jianan Wang, Qinghuai Liu, Ping Xie

**Affiliations:** Department of Ophthalmology, The First Affiliated Hospital of Nanjing Medical University, No. 300, Guangzhou Road, Nanjing, Jiangsu 210029 People’s Republic of China

**Keywords:** Internal limiting membrane, Flap, Macular hole, Inner retinal dimplings

## Abstract

**Background:**

Internal limiting membrane (ILM) peeling increases the idiopathic macular hole (IMH) closure rate but causes the inner retina dimplings. This study is to introduce a method to minimally peel the ILM, and with the ILM flap to ensure the IMH closure.

**Methods:**

Twelve consecutive IMH eyes were treated with the minimal ILM peeling with ILM flap technique. The ILM around the MH is peeled off in an annular shape with a width of approximately 200 to 300 μm. A tongue-shape ILM flap is created in the superior retina and the inferior margin of ILM is not peeled off. The ILM flap is then inverted to cover the MH, followed by fluid-air exchange and air or silicon tamponade. Spectral domain-optical coherence tomography (SD-OCT) and *en face* OCT for morphological assessment, best corrected visual acuity (BCVA) and multifocal electroretinogram (ERG) for functional evaluation were performed at baseline and at each postoperative follow-up.

**Results:**

All the 12 eyes achieved macular hole closure on SD-OCT after surgery (100%). At baseline, the mean preoperative BCVA was 0.83 ± 0.33 and it improved to 0.39 ± 0.28 postoperatively (*p* <  0.001). *En face* OCT showed the inner retinal dimplings were localized only in superior ILM-free retinas (7 eyes). The mERG response density in the central (R1), para-central (R2), R1/R2 ring ratios were remarkably improved at the last follow-up (*p* = 0.001, *p* = 0.033, *p* = 0.018, respectively).

**Conclusions:**

The minimal ILM peeling with ILM flap technique can achieve favorable MH closure with less inner retinal dimplings and has promising visual recovery for IMH eyes.

## Background

Internal limiting membrane (ILM) peeling has been accepted as one of the standardized procedures in idiopathic macular hole (IMH) surgery. Although ILM peeling is favorable for macular hole closure [1], especially for those with large hole sizes, it may also cause side-effects on retinal microstructure and function. The reported retinal microstructure abnormities after ILM peeling include inner retinal dimplings [2], dissociated retinal nerve fiber layer (RNFL) [3], and reduced parafoveal retinal thickness [4].

With the application of *en face* optical coherence tomography (OCT), inner retinal dimplings has been appreciated, corresponding to ganglion cell RNFL thinning [2, 5, 6]. Though the development of inner retinal dimplings is poorly understood, it has been indicated that it may be due to injury of the Müller cell footplates [2, 7, 8]. The visual recovery after IMH surgery is associated with the integrity of retinal microstructure such as inner retinal dimplings, external limiting membrane (ELM), ellipsoid zone (EZ), and retinal pigment epithelium status [9–11]. Using the focal macular electroretinogram, Terasaki et al [12] found that there was delayed and limited B-wave recovery in the first 6 months after ILM peeling for patients with IMH.

To avoid or minimize the damage of retinal microstructure by ILM peeling, some surgeons introduced new techniques aiming to preserve the ILM for IMH [13, 14]. Of note, new techniques can only be induced in clinical practice and on condition of not compromising the IMH closure rate. On the other hand, the inverted ILM flap technique, first reported by Michalewska et al [15], has been demonstrated to improve the closure rate for those large or chronic IMHs. It is hypothesized that the inverted ILM flap may provide a scaffold for tissue proliferation.

Here, in consideration of adverse effects of routine ILM peeling, the authors describe a minimal ILM peeling method with ILM flap technique for IMH eyes in order to preserve the ILM and ensure the IMH closure rate.

## Methods

### Study design and patients

This was a prospective, consecutive, interventional preliminary study conducted in the First Affiliated Hospital of Nanjing Medical University. This study was performed according to the Declaration of Helsinki and Tokyo for humans, and approved by Ethic Committee of First Affiliated Hospital of Nanjing Medical University (approval number: 2015-SR-191). Written informed consent was also obtained from all participants.

From April 01, 2019 to May 15, 2019, 12 eyes of 12 patients who underwent vitrectomy to treat IMH were included in the study. According to the Gass classification system [16], patients diagnosed with a stage 2, 3, or 4 IMH met with the inclusion criteria. The presence of an macular hole was confirmed by spectral-domain optical coherence tomography model on AngioVue (Optovue, Fremont, CA, USA). Main exclusion criteria were eyes with myopic MH, or traumatic MH, or MH associated with retinal detachment, or eyes combined with choroidal neovascularization, or macular atrophy. Also excluded were patients with a history of other retinal disorders such as severe cataract, diabetic retinopathy, or retinal vein occlusion that could potentially affect the central vision acuity. All of the patients had postoperative follow-up periods of at least 2 months.

### Surgical technique

**Fig. 1:**
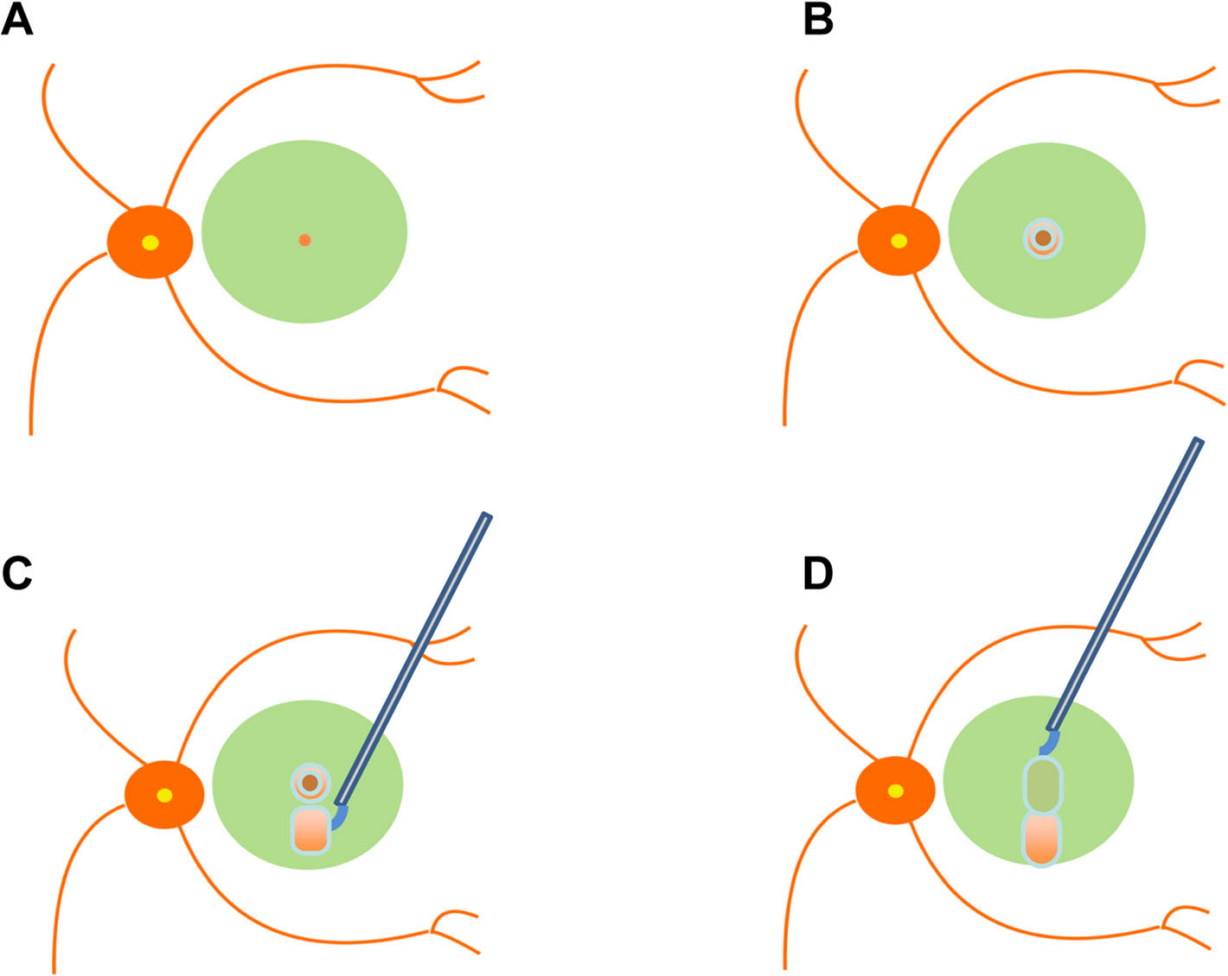
Schematic drawing of step-by-step flow chart of minimal internal limiting membrane peeling with ILM flap technique for idiopathic macular holes. **a**, ILM stain with indocyanine green. **b**, The ILM around the MH of is peeled off in an annular shape with a width of approximately 200 to 300 μm. **c**, A tongue-shape ILM flap is created in the superior retina and the inferior margin of ILM flap is not peeled off. **d**, The tongue-shape ILM flap is inverted to cover the MH and the ILM-free retina

The step-by-step technique of minimal ILM peeling with ILM flap technique for IMH is described in Fig. 1 and video (see Video, **Supplemental file****1**). If there is lens opacity for the patients, phacoemulsification is conducted followed by placement of an intraocular lens. Afterwards, a standard pars plana vitrectomy (PPV) is performed by a single experienced surgeon (P.X) using the 3-port, 23/ 25-gauge vitrectomy system (Alcon Laboratories, Inc., Fort Worth, TX) under a noncontact viewing system Resight 700 (Carl Zeiss Meditec AG, Jena, Germany). The main surgical procedure is as followed:

1. In cases without posterior vitreous detachment (PVD), the PVD is created by suction with the vitrectomy cutter and the posterior hyaloid is thoroughly removed using triamcinolone assisted visualization.
2. The ILM is stained using 0.1 mL of indocyanine green (ICG, 1.25 mg/mL, Eisai, Inc., Shenyang, China) dye for 15–30 s and followed with an immediate lavage.
3. The ILM around the MH of is peeled off in an annular shape with a width of approximately 200 to 300 μm.
4. A tongue-shape ILM flap is created in the superior retina but the inferior margin of ILM flap is not peeled off. The width of the ILM flap is determined to ensure the inverted ILM can cover both side of the ILM-free retina around the MH.
5. The edge of the tongue-shape ILM flap is grasped and then inverted to cover the MH and the ILM-free annular shape retina around MH.
6. Scleral-depressed examination of the periphery retina is performed to identify any retinal breaks.
7. Air-fluid exchange is performed with the air pressure set at 35 mmHg, using a 23/25-gauge flute needle held inferior to the MH and away from the ILM flap.
8. A drop of viscoelastic is introduced to stabilize ILM flap.
9. Finally, the vitreous cavity is filled with air (after step 6) or silicon oil. Patient is then suggested to maintain facedown position for 3 (air as tamponade for IMH size ≤650 μm) or 7 days (silicon oil as tamponade for IMH size > 650 μm).

### Examinations

The primary outcome we measured was the MH closure after surgery on OCT images. The secondary outcomes included the occurrence of inner retinal dimplings on *en face* OCT images, best corrected visual acuity (BCVA), and multifocal ERG response density.

For all patients, the baseline data we collected were a complete record of medical and ophthalmic history, measurement of BCVA, examinations of slit lamp biomicroscopy and binocular indirect ophthalmoscopy, intraocular pressure, axial length assessment using an IOL Master (Carl Zeiss Meditec, Dublin, Calif., USA), macular OCT B-scans with AngioVue (Optovue, Fremont, CA, USA), and multifocal ERG (Roland-RETI scan system, Roland Consult, Brandenburg, Germany). BCVA, *en face* OCT, and multifocal ERG were mainly followed at least 2 months after the surgical intervention.

We used Snellen charts to measure BCVA. BCVA was converted to logarithm of minimum angle of resolution (logMAR) for further statistical analysis.

Radial B-scans (12 lines) and 3D wide-field *en face* methods were used to scan the macula to detect any retinal dimplings.

The multifocal ERG was carried out under the guidelines of the International Society for Clinical Electrophysiology for Vision (ISCEV) 2007 edition [17]. The stimulation source was a monitor with a 75-Hz frame rate. Stimulation calibrations were performed as provided by the RETI-scan software (Roland Consult). The cut-offs were 5 Hz and 100 Hz for the high and low-pass, respectively. The artefact level was 10%.

### Statistical analysis

SPSS software version 20.0 (SPSS, Inc., Chicago, IL) was used to perform statistical analyses of the data. For continuous variables that did not follow normal distribution (logMAR of BCVA, mERG response), wilcoxon signed-rank test was used to compare those values before and after surgery (data on latest follow-up). P value < 0.05 was considered statistically significant.

## Results

To date, we have performed the technique on 12 eyes with IMH in 12 consecutive patients. The preoperative baseline characteristics of all cases are shown in Table 1. The patient population consisted of 8 women (75%) of and 4 men, with a mean age of 62.83 ± 8.64 years (48–77 years). The main macular hole size was 564.67 ± 262.28 μm.

**Table 1:** Demographic data of patients

Number	Age/Sex/Eye	axial length (mm)	Macular hole size (μm)	Tamponade	Macular hole closure	Retinal dimplings	Follow-up (months)
1	69/F/OS	24.5	547	Air	Yes	No	7
2	77/M/OS	23.8	740	SO	Yes	Yes	7
3	48/M/OD	25.2	918	SO	Yes	Yes	6
4	66/F/OS	24.6	188	Air	Yes	No	6
5	68/M/OD	24.3	459	Air	Yes	Yes	6
6	55/F/OD	24.8	801	SO	Yes	No	6
7	64/F/OS	23.4	912	SO	Yes	No	5
8	73/M/OS	24.6	506	Air	Yes	Yes	5
9	62/F/OS	23.5	188	Air	Yes	No	5
10	53/F/OD	23.7	694	SO	Yes	Yes	4
11	64/F/OS	23.5	235	Air	Yes	Yes	3
12	55/F/OD	24.1	588	Air	Yes	Yes	2

*F* female, *M* male, *OD* right eye, *OS* left eye, *SO* silicon oil

**Fig. 2:**
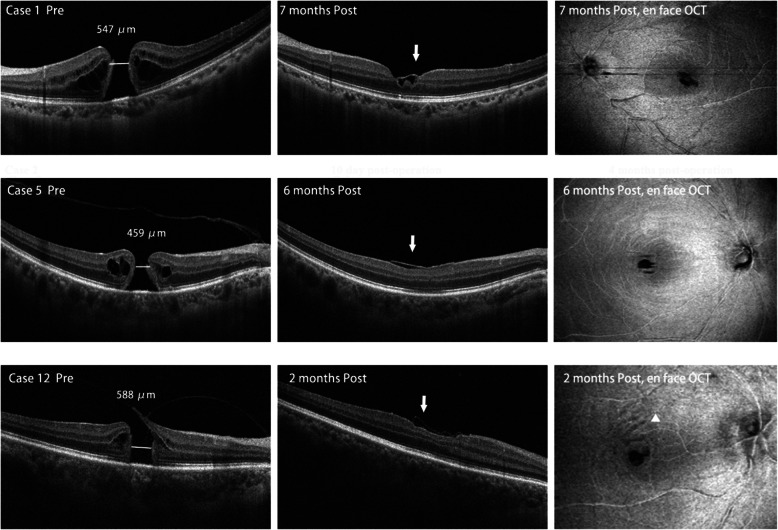
Representative B-scans and wide-field *en face* scans of SD-OCT before and after surgery. After surgery, MH closure is obtained in three representative cases. OCT scanning clearly showed a thin layer structure over the fovea in the 3 eyes, indicating the inverted ILM was not dislocated (arrow). Inner retinal dimplings were observed in local ILM-free retinas with *en face* mode (tringle) in case 12

Successful MH closure was obtained in all cases. No intraoperative or postoperative complications occurred with the new technique. All cases achieved quick macular hole closure on SD-OCT within 1 week and no reopenning during follow-ups after surgery. Inner retinal dimplings were observed in local ILM-free retinas with *en face* mode in 7 eyes (58.3%) at the end of follow-up (Fig. 2). The postoperative OCT scanning also clearly showed a thin layer structure over the fovea in 8 eyes, indicating the inverted ILM was not dislocated (Fig. 2). The mean BCVA (logMAR) remarkably improved from 0.83 ± 0.33 at baseline to 0.39 ± 0.28 (*p* <  0.001) after surgery. Significant improvement of the mERG response density was also found after surgery in the central (R1), para-central (R2), R1/R2, R1/R3, R1/R4, and R1/R5 ring ratios. From baseline to last follow-up, mERG response increased with statistical significance in R1 (*p* = 0.001), R2 (*p* = 0.033), and R1/R2 (*p* = 0.018) (Table 2).

**Table 2:** Vision change pre- and post- operation

Number	BCVA (logMAR)	mERG response					
		R1	R2	R1/R2	R1/R3	R1/R4	R1/R5
Pre-operation	0.83 ± 0.33	85.32 ± 38.33	50.10 ± 19.56	1.75 ± 0.52	3.67 ± 1.77	5.70 ± 2.53	10.51 ± 5.43
Post-operation	0.39 ± 0.28	148.26 ± 59.32	69.08 ± 26.46	2.75 ± 1.28	4.72 ± 1.38	9.40 ± 4.83	23.66 ± 20.09
P value^*^	< 0.001	0.001	0.033	0.018	0.188	0.059	0.080

*BCVA* best corrected vision acuity, *logMAR* logarithm of the minimum angle of resolution

* Wilcoxon signed-rank test

## Discussion

Peeling or not peeling the ILM has been frequently discussed over the past decades [1, 4]. Recently, more and more evidence has indicated inner retinal damage after ILM peeling on *en face* SD-OCT [2, 3, 6, 18]. Furthermore, histological findings further have confirmed that ILM specimens contain neuron elements [8]. All these suggest the importance of the ILM integrity. Herein, we developed here a novel method of minimal ILM peeling with inverted ILM flap technique.

Among the hypotheses concerning the pathogenesis of IMH, the most extensively accepted one is that the abnormal longitudinal (or anterior-posterior) traction by the posterior vitreous cortex on the macular fovea causes the hole while the tangential traction by the ILM enlarges the hole afterwards. The macular hole then usually has an “hourglass” shape, with the edge of the hole curled-up on OCT images. In this new technique of minimally peeling the ILM with the aid of gas or silicon oil, the macular traction force is relieved and the detached edge of the macular hole is reattached. In the conventional ILM peeling method, in which a large area of ILM is peeled off, the tangential traction in this technique is not fully relieved. Therefore, we used the ILM flap technique to provide a scaffold to further facilitate the macular hole closure. To date, we have successfully performed this technique in 12 eyes. Retinal dimplings were only observed in local areas of ILM-free retina with *en face* OCT scanning, suggesting that ILM peeling is the main cause of retinal dimplings. In this study, the incidence of retinal dimplings is 58.3% (7/12), similar to the previously reported, 43 to 86% after ILM peeling [18]. Similarly, Tian et al [14] reported the peeled ILM reposition technique, which preserve the integrity of retina, also yielded better microstructural outcomes of inner retina.

In our new technique, attention should be paid during the air-fluid exchange. We suggested that the flute needle should be placed inferior to the MH to avoid the dislocation of ILM flap. For IMHs smaller than 650 μm, the cavity is filled with air while silicon oil should be used for larger IMHs. Patients were instructed to keep a facedown posture in order to maintain the inverted ILM covering the MH. The postoperative OCT scanning clearly showed a thin layer structure over the fovea in 8 eyes, indicating the inverted ILM was not dislocated. For the other four cases, which the thin layer over the fovea could not be identified, we speculate that the inverted ILM might be attached tightly to the macular and OCT cannot show this structure.

We did not perform the vitreous base shaving. Scleral-depressed shaving of the vitreous base is mostly recommended as part of pars plana vitrectomy (PPV) for primary rhegmatogenous retinal detachment [19, 20]. It has been postulated that the vitreous base may form a scaffold for the development of future anterior proliferative vitreoretinopathy (PVR), and that vitreous base contraction may cause new retinal tears and subsequent retinal detachment [21]. However, complications such as PVR or retinal tears are rarely reported and barely occur in clinical practice. In addition, in the ear of microincisional vitrectomy durgery, small gauges with the trocar/cannula system theoretically create less traction on the vitreous base during instrument entry and exit vitrectomy. Therefore, we think shaving of the vitreous base is not necessary for “simple” macular surgery. Finally, scleral-depressed shaving of vitreous base may be associated with iatrogenic retinal breaks, cataract formation, zonular dehiscence and increased risk of intraoperative choroidal hemorrhage [22]. Of note, shaving of vitreous base is recommended for experienced surgeons and for those large macular holes because this procedure allows injection of more gas mixture into the eye and subsequently prolongs the effect of the gas tamponade. It shows that iatrogenic retinal breaks with vitrectomy under air is less than that with the standard vitrectomy under fluid for macular diseases [23]. In our case series, scleral-depressed peripheral examinations were performed at the end of each surgery and no retinal break was identified in each case at the end of the procedure. As reported, the incidence of retinal breaks of macular diseases such as epiretinal membrane (ERM) and MH was low, ranging from 0 to 15.8% [23–29], but with an average of less than 2.0% [23–27]. The 0% rate of complications in our technique may be owing to the non-scleral-depressed shaving of the vitreous base, vitrectomy by only one experienced surgeon (P.X), and the relatively small sample size.

This study has several limitations. Firstly, this was a preliminary study with no comparison, a further prospective and comparative study with more cases, long-term follow-ups, more outcomes (such as microperimetry) will be needed to verify the efficiency of retinal protection between our technique and routine ILM peeling technique. Secondly, the number of the cases included were relatively small. Thirdly, the macular holes we reported ranged widely in size, ranging from 188 to 918 μm. Finally, this technique actually required more manipulations on macular and longer study curve for younger surgeons.

## Conclusions

In conclusion, the new surgical technique is safe and efficient in treating IMHs. By minimally peeling the ILM, most of the ILM around the MH is preserved, which may be favorable for better inner retinal microstructure after surgery. With the inverted ILM technique, the MH can be more easily closed, especially for those with larger hole size. However, more cases with various hole size and longer follow-up are needed to further confirm the safety and efficiency of this new technique.

## Supplementary information

**Additional file 1 Supplemental file 1 (video):** Key surgical steps of minimal internal limiting membrane peeling with ILM flap technique for idiopathic macular holes.

## Data Availability

The datasets used and/or analyzed during the current study are available from the corresponding author on reasonable request.

## Abbreviations

ILM: Internal limiting membrane
IMH: Idiopathic macular hole
SD-OCT: Spectral domain-optical coherence tomography
BCVA: Best corrected visual acuity
ERG: Electroretinogram
RNFL: Retinal nerve fiber layer
ELM: External limiting membrane
EZ: Ellipsoid zone
ICG: Indocyanine green
logMAR: Logarithm of minimum angle of resolution
PVR: Proliferative vitreoretinopathy
ERM: Epiretinal membrane

## References

[1] Chatziralli IP, Theodossiadis PG, Steel DHW (2018). Internal Limiting Membrane Peeling In Macular Hole Surgery; Why, When, And How?. Retina (Philadelphia, Pa).

[2] Spaide RF (2012). Dissociated optic nerve fiber layer appearance after internal limiting membrane removal is inner retinal dimpling. Retina (Philadelphia, Pa).

[3] Hisatomi T, Tachibana T, Notomi S, Nakatake S, Fujiwara K, Murakami Y, Ikeda Y, Yoshida S, Enaida H, Murata T (2017). Incomplete Repair Of Retinal Structure After Vitrectomy With Internal Limiting Membrane Peeling. Retina (Philadelphia, Pa).

[4] Ohta K, Sato A, Senda N, Fukui E (2018). Comparisons of foveal thickness and slope after macular hole surgery with and without internal limiting membrane peeling. Clin Ophthalmol (Auckland, NZ).

[5] Kishimoto H, Kusuhara S, Matsumiya W, Nagai T, Negi A (2011). Retinal surface imaging provided by cirrus high-definition optical coherence tomography prominently visualizes a dissociated optic nerve fiber layer appearance after macular hole surgery. Int Ophthalmol.

[6] Navajas EV, Schuck N, Govetto A, Akil H, Docherty G, Heisler M, Sarunic MV, Sarraf D (2020). En Face Optical Coherence Tomography And Optical Coherence Tomography Angiography Of Inner Retinal Dimples After Internal Limiting Membrane Peeling For Full-Thickness Macular Holes. Retina (Philadelphia, Pa).

[7] Mitamura Y, Ohtsuka K (2005). Relationship of dissociated optic nerve fiber layer appearance to internal limiting membrane peeling. Ophthalmology.

[8] Tari SR, Vidne-Hay O, Greenstein VC, Barile GR, Hood DC, Chang S (2007). Functional and structural measurements for the assessment of internal limiting membrane peeling in idiopathic macular pucker. Retina (Philadelphia, Pa).

[9] Kim K, Kim ES, Kim Y, Yu SY, Kwak HW (2018). Correlation Between Preoperative En Face Optical Coherence Tomography Of Photoreceptor Layer And Visual Prognosis After Macular Hole Surgery. Retina (Philadelphia, Pa).

[10] Kitao M, Wakabayashi T, Nishida K, Sakaguchi H, Nishida K (2019). Long-term reconstruction of foveal microstructure and visual acuity after idiopathic macular hole repair: three-year follow-up study. Br J Ophthalmol.

[11] Reibaldi M, Avitabile T, Longo A, Uva MG, Bonfiglio V, Russo A, Toro MD, Stella S, Giovannini A, Viti F (2014). Correlation of preoperative retinal pigment epithelium status with foveal microstructure in repaired macular holes. Ophthalmol J Int Ophtalmolog Int J Ophthalmol Zeitschrift Augenheilkunde.

[12] Terasaki H, Miyake Y, Nomura R, Piao CH, Hori K, Niwa T, Kondo M (2001). Focal macular ERGs in eyes after removal of macular ILM during macular hole surgery. Invest Ophthalmol Vis Sci.

[13] Hu Z, Ye X, Lv X, Liang K, Zhang W, Chen X, Cao E, Gu X, Liu Q, Xie P. Non-inverted pedicle internal limiting membrane transposition for large macular holes. Eye (London, England). 2018, 32(9):1512–8. 10.1038/s41433-018-0107-2 PMC6137144 29844368

[14] Tian T, Chen C, Peng J, Jin H, Zhang L, Zhao P: Novel surgical technique of peeled internal limiting membrane reposition for idiopathic macular holes. Retina (Philadelphia*,* Pa) 2017. 10.1097/IAE.0000000000001745 28696343

[15] Michalewska Z, Michalewski J, Adelman RA, Nawrocki J (2010). Inverted internal limiting membrane flap technique for large macular holes. Ophthalmology.

[16] Gass JDM (1988). Idiopathic senile macular hole: its early stages and pathogenesis. Arch Ophthalmol.

[17] Hood DC, Bach M, Brigell M, Keating D, Kondo M, Lyons JS, Palmowski-Wolfe AM (2008). ISCEV guidelines for clinical multifocal electroretinography (2007 edition). Doc Ophthalmol.

[18] Amouyal F, Shah SU, Pan CK, Schwartz SD, Hubschman JP (2014). Morphologic features and evolution of inner retinal dimples on optical coherence tomography after internal limiting membrane peeling. Retina (Philadelphia, Pa).

[19] Lai MM, Ruby AJ, Sarrafizadeh R, Urban KE, Hassan TS, Drenser KA, Garretson BR (2008). Repair of primary rhegmatogenous retinal detachment using 25-gauge transconjunctival sutureless vitrectomy. Retina (Philadelphia, Pa).

[20] Martinez-Castillo V, Boixadera A, Garcia-Arumi J (2009). Pars plana vitrectomy alone with diffuse illumination and vitreous dissection to manage primary retinal detachment with unseen breaks. Arch Ophthalmol.

[21] Chaturvedi V, Basham RP, Rezaei KA (2014). Scleral depressed vitreous shaving, 360 laser, and perfluoropropane (C3 F8) for retinal detachment. Indian J Ophthalmol.

[22] Tabandeh H, Sullivan PM, Smahliuk P, Flynn HW, Schiffman J (1999). Suprachoroidal hemorrhage during pars plana vitrectomy. Risk factors and outcomes. Ophthalmology.

[23] Reibaldi M, Rizzo S, Avitabile T, Longo A, Toro MD, Viti F, Saitta A, Giovannini A, Mariotti C (2014). Iatrogenic retinal breaks in 25-gauge vitrectomy under air compared with the standard 25-gauge system for macular diseases. Retina (Philadelphia, Pa).

[24] Patelli F, Radice P, Zumbo G, Frisone G, Fasolino G (2007). 25-gauge macular surgery: results and complications. Retina (Philadelphia, Pa).

[25] Nakano T, Uemura A, Sakamoto T (2011). Incidence of iatrogenic peripheral retinal breaks in 23-gauge vitrectomy for macular diseases. Retina (Philadelphia, Pa).

[26] Cha DM, Woo SJ, Park KH, Chung H (2013). Intraoperative iatrogenic peripheral retinal break in 23-gauge transconjunctival sutureless vitrectomy versus 20-gauge conventional vitrectomy. Graefes Arch Clin Exp Ophthalmol.

[27] Mura M, Barca F, Dell'Omo R, Nasini F, Peiretti E (2016). Iatrogenic retinal breaks in ultrahigh-speed 25-gauge vitrectomy: a prospective study of elective cases. Br J Ophthalmol.

[28] Yu Y, Qi B, Liang X, Wang Z, Wang J, Liu W. Intraoperative iatrogenic retinal breaks in 23-gauge vitrectomy for stage 3 and stage 4 idiopathic macular holes. Br J Ophthalmol. 2020. 10.1136/bjophthalmol-2019-315579 32217539

[29] Tan HS, Mura M, De Smet MD (2009). Iatrogenic retinal breaks in 25-gauge macular surgery. Am J Ophthalmol.

